# A single lateral hinge screw increased resistance to varus stress after medial closing wedge distal femoral osteotomy in a Sawbones model: A biomechanical analysis

**DOI:** 10.1002/jeo2.70566

**Published:** 2025-11-18

**Authors:** Benjamin J. Main, Matthieu Ollivier, Pooyan Abbasi, Steven J. Svoboda, James C. Dreese, Jingyi Shao, Wiemi A. Douoguih

**Affiliations:** ^1^ MedStar Lafayette Orthopaedic and Sports Medicine Center MedStar Washington Hospital Center Washington DC USA; ^2^ Institut du Mouvement et de l'Appareil Locomoteur, Hospital Sainte‐Marguerite Aix‐Marseille Universite Marseille France; ^3^ Department of Orthopaedic Surgery MedStar Union Memorial Hospital Baltimore Maryland USA; ^4^ Department of Orthopaedic Surgery MedStar Georgetown University Hospital Washington DC USA; ^5^ Department of Orthopedic/Sports Medicine Research MedStar Health Research Institute Columbia Maryland USA

**Keywords:** distal femoral osteotomy, hinge fracture, lateral hinge fracture, osteotomy

## Abstract

**Purpose:**

To determine the biomechanical effects of a lateral hinge screw on postoperative fracture after medial closing wedge distal femoral osteotomy (MCWDFO). It was hypothesized that adding a lateral hinge screw would significantly increase the failure strength of the hinge.

**Methods:**

Twelve Sawbone femurs were used for the study. A biplanar cut was made, a protective lateral hinge pin was placed, and a 5 mm‐closing wedge osteotomy was performed in each specimen. After osteotomy, the hinge pin was removed in six specimens, and in the remaining six specimens, the hinge pin was replaced with a lateral hinge screw. A varus load was then applied utilizing a load frame in a single load‐to‐failure test. Failure was defined as the point just before a substantial drop in the force versus displacement curve. Nonparametric Wilcoxon rank‐sum test was performed to compare maximum load and stiffness between the hinge screw and no hinge screw groups with *p* < 0.05 considered the threshold of statistical significance.

**Results:**

The maximum load to failure was significantly higher in the hinge screw group versus the control group, 440 ± 70 N versus 256 ± 107 N (mean ± standard deviation), respectively. Load to failure was 72% higher in the hinge screw group versus the no hinge screw group. No significant difference in stiffness was found between the groups.

**Conclusion:**

Using a Sawbone model, the current data showed that placement of a lateral hinge screw significantly increased resistance to varus stress following MCWDFO compared with a construct having no screw. Additionally, no significant difference in stiffness was observed between the group with the hinge screw and the group without the screw. Further investigation should assess the clinical benefit of a hinge screw in reducing surgical morbidity during the postoperative period for patients undergoing a distal femoral osteotomy.

**Level of Evidence:**

N/A.

## Graphical abstract

In a Sawbone model, placement of a lateral hinge screw significantly increased resistance to varus stress after medial closing wedge distal femoral osteotomy compared with a construct having no screw.

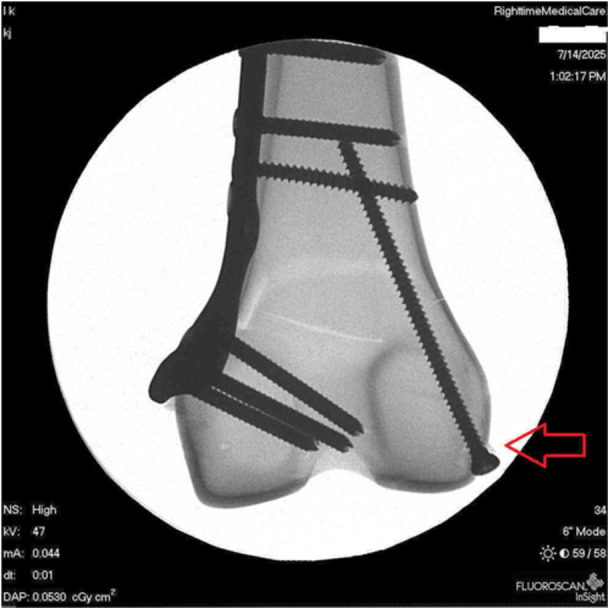

**Abbreviations**
DFO: distal femoral osteotomy
IQR: interquartile range
LOWDFO: lateral opening wedge distal femoral osteotomy
MCWDFO: medial closing wedge distal femoral osteotomy
SD: standard deviation

## INTRODUCTION

Distal femoral osteotomy (DFO) is a well‐established technique to treat malalignment of the knee in a number of clinical scenarios, including recurrent patellar instability, a symptomatic compartment in the setting of articular cartilage and meniscus repair, and knee pain from unicompartmental osteoarthritis. DFO surgery redistributes load from the diseased arthritic compartment of the knee to the unaffected tibiofemoral knee compartment. Hinge fracture after DFO is a major complication and can result in delayed healing and loss of correction [5, 10, 12]. Up to 60% of DFO surgeries may have a hinge fracture, and many of these fractures are missed [20].

The stabilizing effect of a hinge screw has been previously evaluated in DFO when compression and rotation forces were placed on the construct [2, 16, 18, 21, 26], but not with a varus load applied to the knee. It would be useful to understand the effect of a prophylactic lateral hinge screw when performing medial closing wedge distal femoral osteotomy (MCWDFO) to prevent or mitigate the effects of a varus stress applied to the lateral hinge. A prior Sawbones study showed the positive effect of a lateral hinge screw after high tibial osteotomy in preventing fracture of the lateral tibial hinge with varus stress in a Sawbones model [7].

Our objective was to compare the lateral hinge fracture failure load with varus load after MCWDFO with and without the use of a lateral hinge screw. It was hypothesized that the use of a lateral hinge screw would result in significantly higher failure load compared with no lateral screw after MCWDFO.

## METHODS

A synthetic bone model (Sawbones USA, fourth‐generation 17 PCF composite femur) was utilized for reproducibility and standardization [7, 26]. The specimens were identical and were numbered for testing purposes. All procedures were performed by a single, fellowship‐trained orthopaedic surgeon.

### Surgical technique

**Figure 1:**
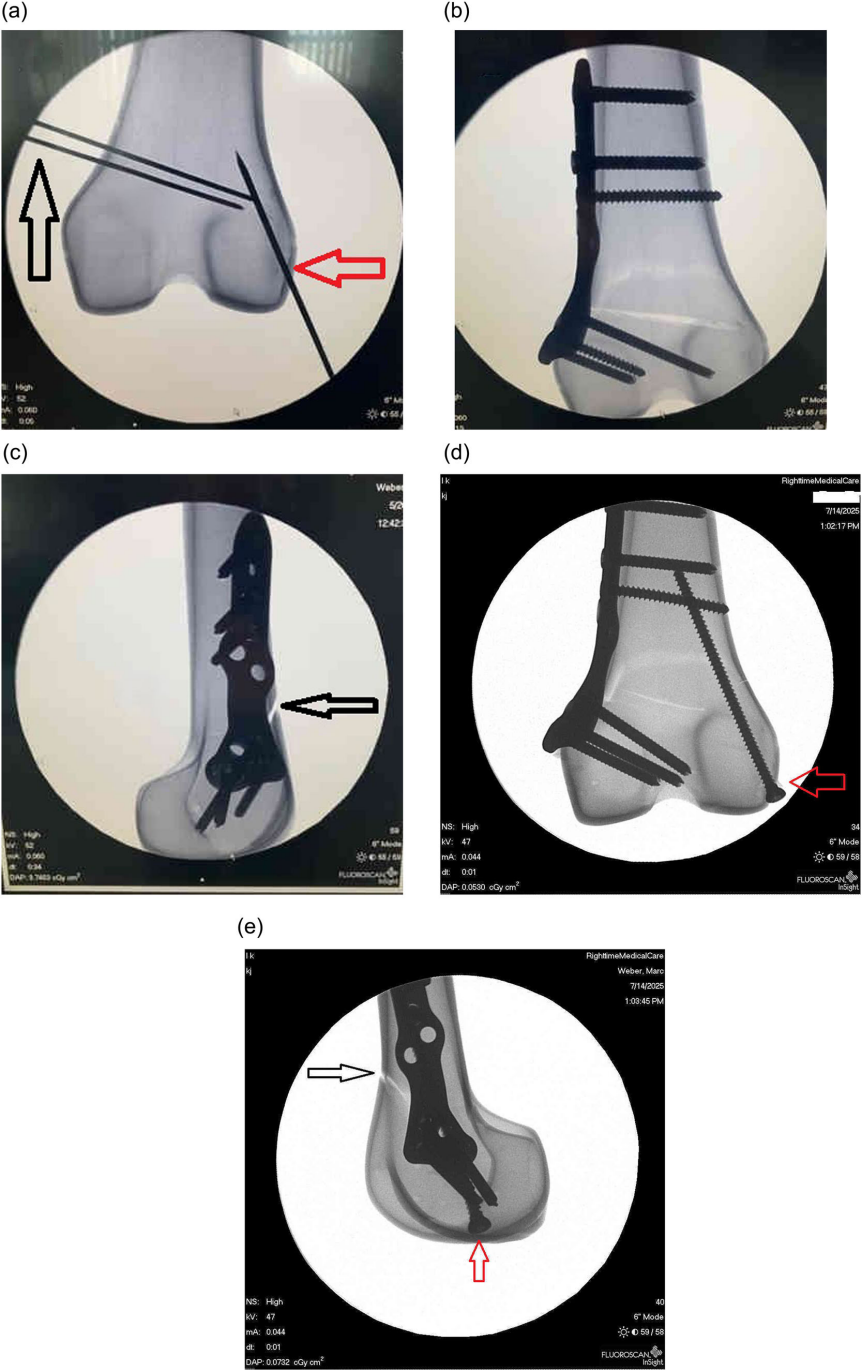
Fluoroscopy images of femoral Sawbone specimens. (a) Guide pins for distal femoral osteotomy (DFO) indicated (black arrow) and hinge wire (red arrow). (b) Plate without hinge screw. (c) Lateral fluoroscopic image of DFO with plate and biplanar cut (black arrow). (d) Anteroposterior fluoroscopic view of DFO with hinge screw (red arrow). (e) Lateral fluoroscopic image of DFO with biplanar cut (black arrow) and hinge screw (red arrow).

The Sawbones were prepared by performing a standard MCWDFO with a biplanar cut. The biplanar cut is used routinely in our clinical practice to optimize stabilization of the osteotomized bony surfaces (Figure 1). Using a biplanar technique has been shown to better control rotation in the axial and sagittal planes and provides more surface area for healing [5, 24, 25]. Under fluoroscopic guidance, two 1.6‐mm Kirschner wires were placed in the medial distal femur. The pins were 5 mm apart on the medial cortex with a slight convergence with the distal pin placed 4.5 cm from the medial condyle. Under fluoroscopic guidance, a 2.2‐mm Kirschner wire was then inserted on the inferolateral cortex parallel to the lateral cortex of the femur 10 mm from the joint line running 10 mm medial to the lateral cortex. The 2.2‐mm wire acted as the hinge wire and was used to limit the cut depth and help preserve the lateral hinge during osteotomy opening to prevent hinge fracture [9, 14].

A 2‐cm Stryker Performance series blade (Stryker) and fluoroscopy were used during the planned osteotomy. The inferior axial osteotomy cut was made, following the inferior Kirschner wire. Then the sagittal biplanar cut was made at 110° to the axial cut. The superior axial cut was then made following the superior Kirschner wire. The 1.6‐mm Kirshner wires were removed along with the Sawbone wedge, and the osteotomy site was opened 5 mm for each specimen. The medial osteotomy site was closed manually, and an MCWDFO osteotomy locking plate (Activmotion S precontoured, Newclip Technics) was placed. Two bicortical nonlocking screws were placed through the plate, one superior and one inferior to the osteotomy site in compression mode. Locking screws were then placed, two above and two below the osteotomy site.

After the osteotomy was performed and secured with a plate, the lateral hinge wire was removed. The Sawbone specimens were identical, and therefore no randomization was required. Six of the 12 specimens received a hinge screw. A single 3.5‐mm cortical screw was placed using a lag technique in the same tract as the 2.2‐mm hinge wire. The screw was placed parallel to the lateral femoral cortex at the lateral hinge under fluoroscopic guidance. The distal femur epiphysis of each specimen was embedded into a resin mould (Bondo Filler, 3M) with 2‐mm Kirschner wires inserted through the casing and tibial epiphysis parallel to the osteotomy.

### Biomechanical testing

**Figure 2:**
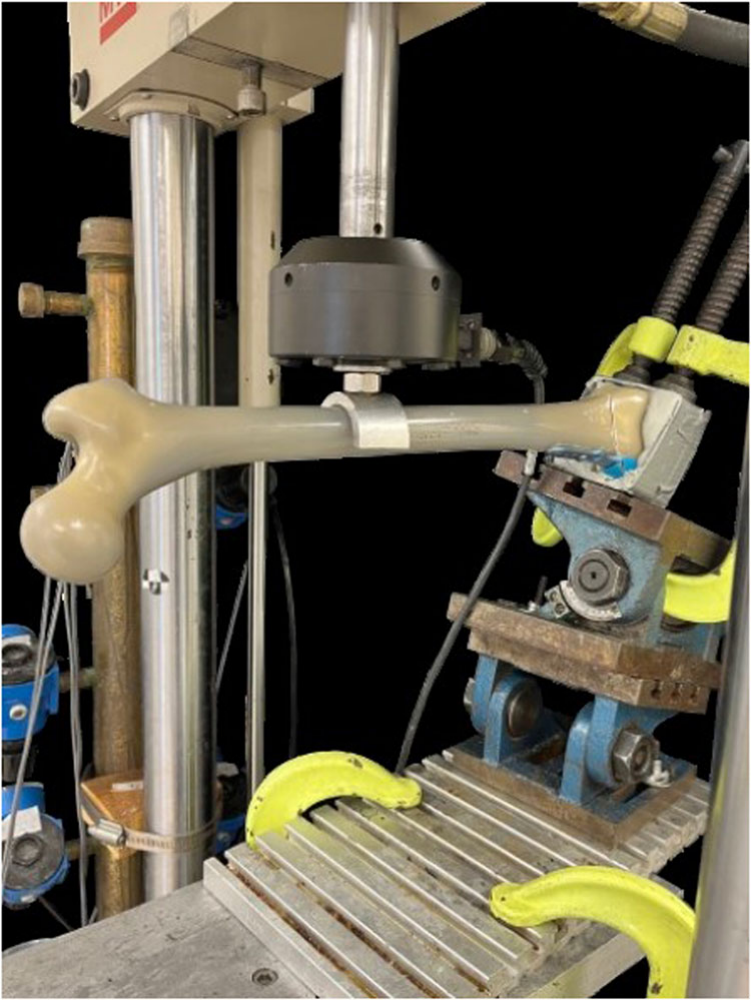
Biomechanical testing setup showing load frame for single load‐to‐failure test. Load was applied utilizing the 858 Mini Bionix load frame in a single load‐to‐failure test. Two Swivel angle tables were used to align the specimens with the load frame for varus loading.

The distal femur was fixed, and a varus load was applied at a standardized distance along the diaphysis to reproduce a varus load. Load was applied utilizing a MTS 858 Mini Bionix load frame (accuracy 0.1% of applied load based on annual sensor calibration by the manufacturer) in a single load‐to‐failure test. Two perpendicular angle plates were used to align the samples with the load frame for varus loading (Figure 2). The presence of an intact lateral distal femur hinge was visually confirmed for each specimen before testing. Quasistatic loading with prescribed displacements at 10 mm/min was applied until failure, based on previously published protocols [9, 23]. Vertical displacement and applied load were recorded. Failure was defined as the point of a substantial drop in the force versus displacement curve. Force at failure (maximum load) and stiffness were identified. Location of failure was noted.

### Statistical analysis

Wilcoxon rank‐sum tests were used to compare maximum load and stiffness between hinge screw and nonhinge screw groups with *p* < 0.05 considered the threshold of statistical significance. Median/interquartile range (IQR) is appropriate when reporting the results of nonparametric analysis. However, units of mean/standard deviation (SD) were retained because of the small sample size. A priori power analysis was performed based on a previous study [7] comparing load to failure with and without a lateral hinge screw with an SD of 82.8 N and an effect size of 2.22. Based on these data, six specimens were needed in each group for 90% power to detect a significant difference with a significance level of 0.050.

## RESULTS

The results for all specimens are shown in Table 1. The maximum load to failure was significantly higher in the hinge screw group compared to the control group, 440 ± 70 N versus 256 ± 107 N (mean ± SD), respectively (*p* = 0.026). Load to failure was 72% higher in MCWDFO using a hinge screw versus no hinge screw. There was no significant difference in stiffness between the groups (*p* = 0.937; Table 2). Location of failure for each specimen is summarized in Table 3.

**Table 1:** Specimen‐level maximum force and stiffness by group.

Group	Specimen no.	Maximum force (N)	Stiffness (N/mm)
Without screw	1	266.1	14.7
	2	227.8	16.2
	4	203.6	24.7
	5	153.9	35.5
	10	461.5	38.2
	12	221.0	37.4
With screw	3	554.6	10.3
	6	428.3	6.7
	7	391.8	40.3
	8	403.8	38.7
	9	367.8	19.3
	11	491.9	24.7

Abbreviations: mm, millimetres; N, Newtons.

**Table 2:** Summary of testing results.

Parameter	Hinge screw	No hinge screw	Mean difference	Absolute difference	*p* value
Load to failure (N)	439.7 ± 70.5	255.6 ± 107.3	184.1	+72%	0.026
Stiffness (N/mm)	23.3 ± 14.1	27.8 ± 10.7	−4.5	−16%	0.937

Abbreviations: mm, millimetres; N, Newtons.

**Table 3:** Location of failure in test specimens.

Group	Specimen no.	Failure location
Without screw	1	Lateral side fractured, aligned with the anterior and the posterior cuts
	2	Lateral side fractured, aligned with the anterior and the posterior cuts
	4	Lateral side fractured, aligned with the anterior cut, superior to the posterior cut
	5	Lateral side fractured, aligned with the anterior and the posterior cuts
	10	Lateral side fractured, aligned with the anterior cut, superior to the posterior cut
	12	Lateral side fractured, aligned with the anterior and the posterior cuts
With screw	3	Lateral side fractured, aligned with the anterior cut, superior to the posterior cut
	6	Lateral side fractured, aligned with the anterior cut, superior to the posterior cut
	7	Lateral side fractured, aligned with the anterior cut, superior to the posterior cut
	8	Lateral side fractured, aligned with the anterior cut, superior to the posterior cut
	9	Lateral side fractured, aligned with the anterior cut, superior to the posterior cut
	11	Lateral side fractured, aligned with the anterior and the posterior cuts

## DISCUSSION

In this biomechanical Sawbones model, the placement of a lateral hinge screw resulted in significantly higher load to failure with varus stress testing after MCWDFO compared with an MCWDFO construct that did not include a lateral hinge screw. Load to failure of the lateral hinge in the lateral hinge screw group was 72% higher than in the no screw group. Given the high rate of hinge fracture after MCWDFO and associated morbidity, it would be useful to have a comprehensive understanding of the potential benefits of a lateral hinge screw for risk mitigation.

The significantly higher load to failure with varus stress testing after MCWDFO suggests that a lateral hinge screw construct could help prevent lateral hinge fracture after MCWDFO and its associated morbidity. Furthermore, the finding of increased failure load without a significant difference in stiffness suggests that the screw adds strength but would not interfere with bone healing. An excessively stiff construct has been shown to adversely affect the healing process [1, 4].

Despite excellent outcomes with DFO overall [3, 5, 8, 10, 20, 25], hinge fracture can compromise the stability of DFO and lead to pain, delayed healing, loss of correction, malunion and nonunion. A study by Nha et al. detected a lateral hinge fracture in 60% of patients undergoing MCWDFO [19]. In a study of 21 patients undergoing MCWDFO, Fujita et al. found a 57% incidence of lateral hinge fracture. In the presence of a hinge fracture, a healing rate >3 months was found in 67% of patients with lateral hinge fracture and in only 11% of patients when a hinge fracture was not present. Of the nine patients with slow healing, six exhibited an average correction loss of 4.3° [11].

With the burgeoning interest in knee osteotomy, efforts to identify risk mitigation strategies are essential to optimize patient outcomes. MCWDFO has gained increasing attention in the United States over the last several years. It offers advantages over lateral opening wedge distal femoral osteotomy (LOWDFO) to treat lateral unicompartmental osteoarthritis because MCWDFO has less effect on patellar lateralization, less iliotibial band lengthening and pain, less knee stiffness and inherently greater stability compared with the lateral opening wedge procedure [17, 19, 21, 24, 25]. Measures to help prevent lateral hinge fracture would further add to the list of potential benefits of MCWDFO.

Two previous biomechanical studies showed increased construct stability with placement of a lateral hinge screw for hinge fracture fixation during MCWDFO [18, 21]. A 2022 study by Matsushita and colleagues used a cadaveric limb model to assess lateral hinge fracture versus no hinge fracture after monoplanar MCWDFO [18]. Translation, rotation and strain were significantly lower in the group with the lateral hinge fracture fixed with a lateral plate compared to the group with no hinge fracture. The addition of a lateral hinge plate reduced motion and strain by 60%. Peez et al. assessed fixation of hinge fractures for both monoplanar and biplanar LOWDFO and MCWDFO [21]. The study showed that the presence of a hinge fracture significantly increased rotational displacement and reduced stiffness. Plate and screw fixation improved resistance to compression and rotation of the hinge fracture, with plate fixation displaying the highest stiffness and least displacement for MCWDFO and LOWDFO. Although both of those studies showed improved stability when the lateral hinge was internally fixed, neither study addressed the important effect of coronal plane stresses that may occur postoperatively.

Previous studies evaluating the lateral hinge have tested the hinge in compression and rotation [18, 21]. Although a lateral hinge fracture has been shown to destabilize the DFO construct, compression and rotation may stabilize rather than stress the hinge in a MCWDFO construct [7, 18, 21]. Hinge fractures are created by weakening of the bone in the varus–valgus plane. Varus loading was chosen because there is a significant knee adduction moment in normal gait (0.1 to +0.4 mm/N) [15]. Varus loading represents a more likely postoperative clinical scenario that would put the construct at risk. Varus load can occur as a result of normal gait, awkward gait patterns due to pain, stumbles and falls after surgery [7]. Further clinical studies will be needed to better elucidate the relationship between our findings and the in vivo loading forces after osteotomy.

The present study had several limitations. This was a biomechanical study in a Sawbone model, so the surrounding soft tissue and the cellularity of bone could not be considered in our testing. Additionally, the inability of Sawbones to fully reproduce the physiologic properties of human bone, such as overall modulus of elasticity and differential in stiffness between cancellous and cortical bony surfaces, could limit extrapolation of the findings to clinical scenarios. However, despite this limitation, several studies have utilized Sawbones for biomechanical testing of bone/plate constructs. Because Sawbones are identical, investigators can reproducibly perform osteotomies and place plates and screws using identical landmarks on each specimen. This level of consistency may be more difficult to achieve in cadavers because of inherent heterogeneity and composition [6, 7, 13, 22, 26]. Load to failure was assessed using a varus force. Although varus forces are a component of normal ambulation, postoperative human gait involves more complex loading patterns. These concerns notwithstanding, varus loads are an important part of the human gait cycle and the lateral hinge is the most vulnerable region of the proximal tibial osteotomy site with respect to varus stress postoperatively. In addition, screw versus no screw was not evaluated in a hinge fracture model. This was done explicitly for proof of concept with an intact hinge. Future studies should investigate screw versus no screw constructs in the presence of a hinge fracture. Another limitation was the high load to failure observed in this study. Although this value may not be clinically relevant for normal gait, the loads experienced during falls and awkward landings may exceed the values found in this study.

## CONCLUSION

Using a Sawbones model, the current data showed that placement of a lateral hinge screw significantly increased resistance to varus stress following MCWDFO compared with a construct having no screw. Additionally, no significant difference in stiffness was observed between the hinge screw and the no screw group.

## AUTHOR CONTRIBUTIONS

All authors contributed to the study conception and design. Material preparation and data collection were performed by Benjamin J. Main, Matthieu Ollivier, Pooyan Abbasi and Wiemi A. Douoguih. Analysis was performed by Jingyi Shao. The first draft of the manuscript was written by Benjamin J. Main, and all authors commented on previous versions of the manuscript. All authors read and approved the final manuscript.

## CONFLICT OF INTEREST STATEMENT

M.O. and W.A.D. are consultants for New Clip Technics. The remaining authors declare no conflicts of interest.

## ETHICS STATEMENT

Our institutional review board does not review cadaveric or synthetic bone studies because they do not meet the criteria for human research studies.

## Data Availability

Data for this study are available upon request.
